# Signaling Mechanism of Transcriptional Bursting: A Technical Resolution-Independent Study

**DOI:** 10.3390/biology9100339

**Published:** 2020-10-19

**Authors:** Yaolai Wang, Jiaming Qi, Jie Shao, Xu-Qing Tang

**Affiliations:** School of Science, Jiangnan University, Wuxi 214122, China; jmq200831@126.com (J.Q.); 1131180207@stu.jiangnan.edu.cn (J.S.); txq5139@jiangnan.edu.cn (X.-Q.T.)

**Keywords:** transcriptional bursts, transcription regulation, cell signaling, stochasticity of gene expression, noise, burst cluster

## Abstract

**Simple Summary:**

Following changing cellular signals, various genes adjust their activities and initiate transcripts with the right rates. The precision of such a transcriptional response has a fundamental role in the survival and development of lives. Quite unexpectedly, gene transcription has been uncovered to occur in sporadic bursts, rather than in a continuous manner. This has raised a provoking issue of how the bursting transmits regulatory signals, and it remains controversial whether the burst size, frequency, or both, take the role of signal transmission. Here, this study showed that only the burst frequency was subject to modulation by activators that carry the regulatory signals. A higher activator concentration led to a larger frequency, whereas the size remains unchanged. When very high, the burst cluster emerged, which may be mistaken as a large burst. This work thus supports the conclusion that transcription regulation is in a “digital” way.

**Abstract:**

Gene transcription has been uncovered to occur in sporadic bursts. However, due to technical difficulties in differentiating individual transcription initiation events, it remains debated as to whether the burst size, frequency, or both are subject to modulation by transcriptional activators. Here, to bypass technical constraints, we addressed this issue by introducing two independent theoretical methods including analytical research based on the classic two-model and information entropy research based on the architecture of transcription apparatus. Both methods connect the signaling mechanism of transcriptional bursting to the characteristics of transcriptional uncertainty (i.e., the differences in transcriptional levels of the same genes that are equally activated). By comparing the theoretical predictions with abundant experimental data collected from published papers, the results exclusively support frequency modulation. To further validate this conclusion, we showed that the data that appeared to support size modulation essentially supported frequency modulation taking into account the existence of burst clusters. This work provides a unified scheme that reconciles the debate on burst signaling.

## 1. Introduction

In individual eukaryotic cells, transcription universally occurs in episodic bursts [1,2,3,4,5,6,7,8,9,10,11,12]. While a gene is “active”, a group of RNA polymerases successively departs from the promoter and gets into elongation, leading to the release of a number of transcripts. The gene then enters into an “inactive” period without the occurrence of an initiation event. Transcriptional bursting represents a much more disordered way than traditionally thought where transcription initiation events were believed to occur smoothly and quasi-continuously [13,14]. Bursty transcription results in dramatic fluctuations in the number of cellular message RNAs (mRNAs) and proteins. This raises an issue that is fundamental for comprehending transcription regulation and cellular signaling of how the bursting dynamics mediate the regulatory signal with the transcriptional level [15,16,17,18,19].

To characterize transcriptional bursting, the bursting dynamics can be usually depicted by three parameters including (1) burst duration, the persistence time of the active period; (2) magnitude, the number of transcripts initiated during an active period, and (3) frequency, the number of bursts that occur per unit time. The burst duration and magnitude are collectively termed as burst size [5,7,8,20]. In principle, an increase in burst in either size or frequency leads to a higher transcriptional level. However, it remains a controversial issue whether burst size, frequency, or both, are modulated by transcriptional activators [5,7,15,16,21,22,23].

**Figure 1:**
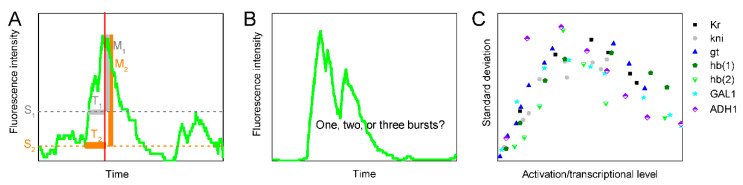
Schematic of technical constraints in quantifying transcriptional bursting. (**A**) The definition of signal detection threshold affects the measured burst size and magnitude. A higher signal detection threshold S1 results in a smaller burst duration (T1) and magnitude (M1) compared to that with a lower threshold (S2). (**B**) When manipulating a pulse with sub-peaks, it is artificial to take it as a single burst or a cluster of overlapping bursts. (**C**) The transcriptional uncertainty data as functions of activation/transcriptional level. Data of eight genes from [31,32,33] are shown. Since the definitions of units in the original studies were different, data from different sources were rescaled for the sake of clarity.

The controversy could be solved if there were no technical resolution constraints. Direct recording of transcriptional bursting is realized by tracing the intensity evolution of the fluorescence that labels nascent transcripts, mature RNAs, or proteins [7,8,24,25,26,27,28,29]. To differentiate and quantify individual bursts in a fluorescence intensity curve, it is inevitable to set two artificial parameters [2,4,7,8,24,25,26,27,28,29,30]. One is the signal detection threshold, which is used to discard the background noise. This threshold affects the measured burst duration and magnitude (Figure 1A). The other parameter is to treat sub-peaks in a “pulse” (Figure 1B). A pulse with two or more sub-peaks may be taken as (1) a single burst where the sub-peaks are treated as fluctuations in the rate of initiating transcripts or (2) two or more neighboring bursts (i.e., a cluster of bursts). That is, both the measured burst size and frequency are not accurate or even mistaken, diminishing the reliability of the results by quantitative measurements.

Ideally, the technical resolution constraint would also be overcome if one could observe how the whole transcription apparatus including the activators, the general transcription factors such as transcription factor IIA (TFIIA), the Mediator, and RNA polymerase II (Pol II) dynamically operate and initiate transcripts at a promoter. However, this remains a challenge far beyond current technologies due to difficulties such as in labeling a huge number of unstable protein complexes [34,35].

Thus, this paper sought to bypass the technical difficulties. For a population of identical cells in homeostasis, transcriptional bursting leads to differences in the number of the gene’s mRNAs or proteins. In turn, the differences reflect the characteristics of transcriptional bursting. Such differences are depicted by the standard deviations from the average. Of note, the measured standard deviation is also a manifestation of other factors such as extrinsic noise and measurement itself. We thus collected those data that mainly reflected intrinsic transcriptional uncertainty rather than extrinsic noise or methodology. As far as we could, we found the data of seven different genes in published papers [31,32,33]. These data are functions of the gene’s average activation level or transcriptional level. Interestingly, all these functions are asymmetrical bell-curves that first increase and then decrease to a value that is larger than the left side (Figure 1C).

We wondered whether the common characteristic of the standard deviation data were capable of inferring the bursting modulation mode. Thus, we introduced two independent theoretical methods. One was an analytical study based on the classic two-state model (also termed as the telegraph model), and the other was an information entropy study based on the general architecture of eukaryotic transcription apparatus. By comparing the experimental data with that predicted by the two methods, the results exclusively support frequency modulation. Moreover, the results unify the other views that appear to be irrelevant. We further demonstrate that the data supporting size modulation actually support frequency modulation, while being aware of the existence of burst clusters.

## 2. Materials and Methods

All the experimental data used were from published papers. As far as we could find, the standard deviation data as functions of activator level or average transcriptional level for seven eukaryotic genes are available [31,32,33]. All these data were collected to compare with our theoretical predictions. The data on burst size and frequency were from [16], where the data were obtained from 8000 promoters of both housekeeping and stimulus-dependent genes. Moreover, the data presented how both the burst size and frequency changed nonlinearly with the increase in transcriptional level in wide ranges. Deductions of the mathematical equations related to the two-state model are available in [36]. Deductions of the other equations are detailed in the main text.

## 3. Results

### 3.1. Analytical Investigations Based on the Two-State Model Support Frequency Modulation

The two-state model is rather simple and analytically tractable. It assumes that gene promoter switches between alternative “ON” and “OFF” states. It has four parameters including activation rate α, namely the transition rate from “OFF” to “ON”, inactivation rate β namely the transition rate from “ON” to “OFF”, mRNA synthesis rate  ε during the “ON” period, and degradation rate δ of mRNA [36,37,38]. α is positively related to the number of transcriptional activators and determines burst frequency. β determines the burst duration, and together with ε determines the burst magnitude. Based on this model, the average number of mRNA M is as follows [36]:

(1) $$M=\frac{\varepsilon }{\delta }\frac{\alpha }{\alpha +\beta }\;,$$

The standard deviation ϕ is as follows:

(2) $$\phi =\sqrt{\frac{\alpha }{\alpha +\beta }\frac{\varepsilon }{\delta }+\frac{\alpha \beta }{{\left(\alpha +\beta \right)}^{2}}\frac{\varepsilon^{2}}{\delta \left(\alpha +\beta +\delta \right)}}\;.$$

The Fano factor F, which is defined as the variance $\phi^{2}$ divided by M, is as follows

(3) $$F=1+\frac{\varepsilon \beta }{\left(\alpha +\beta \right)\left(\alpha +\beta +\delta \right)}.$$

where  F describes the intrinsic noise to gene transcription.

Both ϕ and F are implicit functions of M. By altering α while maintaining the other parameters β, ε, and δ unchanged (i.e., altering bursting frequency while keeping bursting size), it can be obtained how ϕ and F change as functions of M. Similarly, it can be obtained how burst duration or magnitude modulation affect ϕ and F by solely changing β or ε.

**Figure 2:**
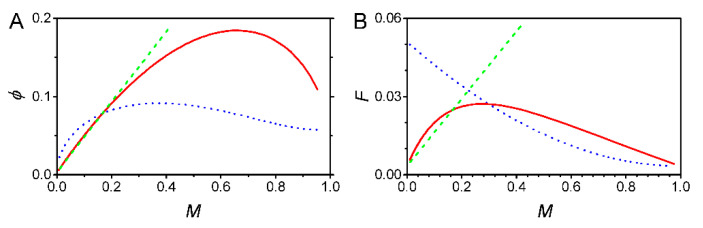
The two-state model intrinsically supports frequency modulation. (**A**) The stand deviation ϕ and (**B**) the Fano factor F as functions of transcriptional level M. Dashed-blue line, frequency modulation; Dashed-green line, magnitude modulation; Red line, duration modulation. The default parameter values are  α  = 0.5 min^−1^, β = 1 min^−1^, ε = 15 min^−1^, and δ = 1/20 min^−1^. All the data were normalized by setting the maximal transcriptional level ε/δ = 1. Changing the values in the biologically reasonable range did not affect the conclusions.

For magnitude modulation, ϕ is a linear function of M (Figure 2A). This is inconsistent with the measured standard deviation. ϕ, corresponding to both duration and frequency modulation, assumes an arched curve that first rises and then decreases (Figure 2A). That is, the curve tendency was similar to that of the experimental data. However, compared with duration modulation, frequency modulation means much smaller uncertainty in a wide range, which benefits the accuracy of cellular signaling. Furthermore, in the range where the transcriptional level is less than ~20% of the maximal transcriptional level, the ϕ of the duration modulation is smaller than that of the frequency modulation; this property substantially affects the Fano factor (Figure 2B). F, corresponding to frequency modulation, is a monotone decreasing function of M, consistent with the common sense that a smaller number of mRNAs means larger fluctuations [21,39,40,41]. In contrast, F, corresponding to duration modulation, is a convex curve that deviates from the common sense. Additionally, the F of magnitude modulation also deviates from the common sense.

In summary, the two-state model experimentally predicts consistent properties of the intrinsic noise, given that transcriptional regulation is achieved via frequency modulation rather than size modulation. Nevertheless, the disadvantages of this investigation are obvious. The two-state model is somehow too simple and the standard deviation curve is not well reproduced. Additionally, it remains to be inferred how the burst frequency encodes the activator level, as this is beyond the two-state model.

### 3.2. Information Entropy Analysis Supports, Reproduces, and Explains Frequency Modulation

The two-state model is too simplistic for an in-depth investigation. We thus introduced the information entropy theory, which naturally characterized the uncertainty of signaling without introducing additional parameters, to study the average uncertainty inherent in transcription regulation. The information entropy S follows:

(4) S=−plnp,

where p is the probability that a transcription initiation event will occur. In the following, we derived the expression of p, based on the general architecture of transcription apparatus revealed by structural and biochemical studies. It should be mentioned that the expression of p, based on a minimal transcription model, was previously obtained [42].

During transcriptional progression, transcriptional activators cyclically bind to and depart from the enhancers [43,44,45,46]. Depending on the current chromatin circumstance, an activator arriving at its enhancer will take different roles [47,48,49]. It may recruit chromatin modifiers to alter epigenetic marks at DNA or histones, resulting in a circumstance suitable for the assembly of transcription apparatus. It may also recruit the components of transcription apparatus to promoter, or catalyze transcription initiation via the Mediator complex.

The general transcriptional factors (GTFs) and RNA polymerase II (Pol II) assemble to the core promoter, leading to the formation of the preinitiation complex (PIC) [50,51,52]. After the Pol II gets into elongation, many GTFs including the Mediator complex remains at the promoter, named after the scaffold complex (SCF), and is sustained for multiple rounds of transcriptional initiations.

**Figure 3:**
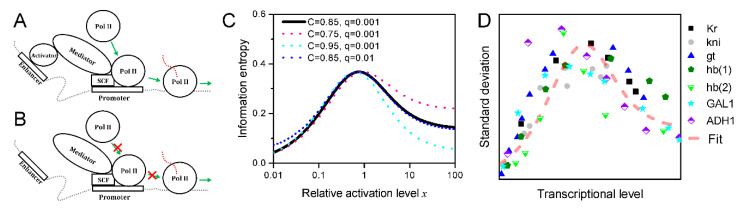
Information entropy of transcriptional regulation based on the architecture of transcription apparatus reproduces transcriptional uncertainty. (**A**) In circumstances where an activator that functions to catalyze transcription initiation is present at the enhancer, successive transcription initiation events lead to a transcriptional burst. (**B**) Else, in the absence of the activator, transcription initiations pause. (**C**) Characteristics of the information entropy as a function of effective activator concentration (i.e., relative activation level). (**D**) Information entropy reproduces the experimental data.

The enhancer-bound activator stimulates transcription initiation via the Mediator complex [51,53]. In the presence of an activator that acts to stimulate transcription initiation via the Mediator complex, Pol IIs successively initiate transcripts one by one with a rate that is certain for a given promoter. This also means that the activator’s dwell time at the enhancer defines the duration of the successive initiation events that constitute a burst [19,26,54] (Figure 3A,B). Additionally, the enhancer-bound activator is dispelled from the enhancer by the transcription apparatus, which is a process independent of activator concentration [44,45,55].

The degree to which the transcription apparatus disassembles and the chromatin structure recovers, before the reconstruction of the transcription apparatus, determines the time interval between transcriptional bursts. In other words, transcriptional bursts are separated by non-transcription intervals affected by many factors such as the stability of SCF and the competition of nucleosomes [2,5].

Based on the general mechanism of transcriptional regulation related above (also shown in the schematic Figure 3A,B), the probability p that a transcription initiation event will occur can be derived. Let A denote a random event that an activator that acts to arouse a transcriptional burst is present at the enhancer. Then,

(5) p=P(E)P(A|E)P(M|A)+q,

where E is the assumed precondition of the occurrence of A and M is the event that a transcript is in gestation. M corresponds to the promoter states that span from the formation of the PIC, the open complex, to the Pol II’s escape. Let P(E)P(M|A)=C, where C can be taken as a constant for a given gene with the concentrations of GTFs and Pol IIs maintaining relative constant. The term q is a constant representing the activator-independent transcription initiation, namely the basal transcription, which is usually very small in eukaryotes.

Considering a period during which activators belonging to A cyclically associate with and depart from the enhancer for m times, the time fraction that the enhancer is bound by the activators follows:

(6) $$R=\frac{\sum_{j=1}^{m}\tau_{on}^{j}}{\sum_{j=1}^{m}\tau_{on}^{j}+\sum_{j=1}^{m}\tau_{off}^{j}},$$

where $\tau_{on}^{j}$ and $\tau_{off}^{j}$ denote the binding time and the unbinding time of the j-th cycle, respectively. By computing the ensemble average of R, it yields:

(7) $$P\left(A|E\right)\overset{p}{\to }\underset{m\to \infty }{\lim}R=\frac{\int_{0}^{1}\frac{1}{a_{off}}\ln\left(\frac{1}{r}\right)dr}{\int_{0}^{1}\frac{1}{a_{off}}\ln\left(\frac{1}{r}\right)dr+\int_{0}^{1}\frac{1}{a_{on}}\ln\left(\frac{1}{r}\right)dr}=\frac{a_{on}}{a_{on}+a_{off}},$$

where r is an independent random number from the uniform distribution in the unit interval, and $a_{on}$ and $a_{off}$ are separately the propensity functions of the activator’s association and disassociation [56,57]. $a_{on}$ is directly proportional to the activator level, whereas $a_{off}$ is a constant for a given gene promoter.

Let $x=a_{on}/a_{off}$, x can be taken as the effective activator concentration. The significance of such a definition is that when x=1, the transcriptional level is half the maximum. Then,

(8) $$P\left(A|E\right)=\frac{x}{x+1}.$$

Substituting Equation (8) to (5), it yields,

(9) $$p=C\frac{x}{x+1}+q.$$

Equation (9) represents a general model that describes the probability a transcription initiation event will occur. Then, the information entropy of transcription regulation follows:

(10) $$S=-\left(C\frac{x}{x+1}+q\right)\ln\left(C\frac{x}{x+1}+q\right).$$

where S as a function of x only involves two parameters C and q, where C predominantly determines the curve tendency whereas q takes an ignorable role (Figure 3C). Recalling that C is the product of P(E) and P(M|A), and both of which are smaller than 1, thus, C must be smaller than 1. On the other hand, to guarantee effective transcriptional regulation, both P(E) and P(M|A) should be as large as possible; otherwise, the regulatory information represented by P(A|E) would be overwhelmed by chaos [42]. With the value of C around 0.85, the common characteristic of transcriptional uncertainty is perfectly reproduced (Figure 3D).

The successful reproduction sources from two points, which relate to the signaling mechanism of transcription regulation. One is the factor P(A|E) that dictates how the activator concentration is encoded by the dynamics of activator–enhancer interactions when the promoter environment allows. The coding mechanism is that the probability of a transcriptional burst to occur is determined by activator concentration. This mechanism determines the main characteristics of transcriptional uncertainty. The other point is a large value of C that guarantees the regulatory information encoded by P(A|E) transmitted with high fidelity. The value of C determines the asymmetry of transcriptional uncertainty.

The signaling mechanism can thus be comprehended in two equivalent ways. The first is, referring to Equations (8) and (9), frequency modulation. The activator level changes burst frequency rather than size. The second is, referring to Equations (6) and (7), the time fraction that the enhancer is occupied by the activators that act to direct transcription initiation. This is essentially the same as the views that explain transcription regulation without the use of “frequency” [32,42].

In conclusion, information entropy analyses connect the architecture of transcription apparatus to transcriptional uncertainty. This suggests that transcription regulation is via burst frequency, which satisfies Equation (9). It also suggests that “the time fraction of enhancer occupation by activators that direct transcription initiation” is an equivalent expression of “frequency modulation”.

### 3.3. Evidence for Size Modulation Intrinsically Support Frequency Modulation

Both investigations above suggest that an increase in activator level leads to an increase in burst frequency, with the burst size maintaining unchanged and the transcriptional level elevated. In the following, we further tested this conclusion with the data that appeared to support size modulation. 

**Figure 4:**
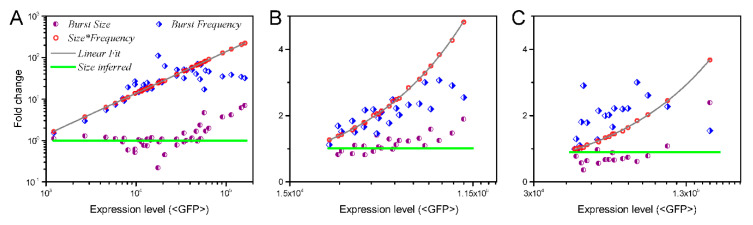
Data supporting size modulation intrinsically support frequency modulation. The measured burst size and frequency of three genes LTR (HIV-1 long terminal repeat), UBC (human elongation factor 1α), and EF1A (human ubiquitin C) are separately presented in panels (**A**–**C**); the data are from [16]. For each gene, the product of burst size and frequency was fitted by a linear function, and the inferred burst size maintained constant.

A quantitative analysis on 8000 individual human genomic loci suggested that both burst size and frequency are altered by transcriptional activators [16]. At low transcriptional level, the increase in transcriptional level is due to the increase of burst frequency, whereas burst size remains nearly unchanged. However, as the expression level increases, the burst size gradually increases, whereas the frequency tends to decrease (Figure 4A–C).

Indeed, frequency modulation explains and reconciles such findings. According to our conclusions, at low transcriptional levels, the probability of a transcription initiation event to occur is low and it is thus easy to differentiate individual bursts. This explains the observation that the burst frequency increases while the burst size remains unchanged. At high transcriptional levels, as the probability of a transcription initiation event to occur becomes high, the intervals between bursts shrinks. Dense bursts means the individuals are no longer easy to differentiate. Instead, due to the resolution constraint, a cluster of bursts is likely to be taken as a single burst, and consequently, the recorded burst frequency decreases. That is, our conclusions explain the conflict.

The explanation above can be quantitatively tested. For a gene that expresses stably, the average number N of its product protein is proportional to its transcriptional rate R. This is because transcriptional elongation and translation do not introduce nonlinear effects [4,33,58]. That is, N=kR, where k is the proportionality coefficient. R=fs+b, where f and s are separately the burst frequency and size, and b denotes a fluctuation due to other factors such as experimental errors and basal transcription. Then, it yields N=kfs+kb. We therefore calculated the product of the measured burst frequency and size, and made a linear fit to the product with the use of fs=N/k−b (Figure 4A–C). Results showed that the product can be perfectly fitted for all three genes. Given that s is a constant, N is also a linear function of f. Since the burst sizes were normalized by setting its value at a low transcriptional level to “1”, the inferred burst size is also shown in Figure 4A–C. This suggests that the data can also be explained by frequency modulation.

In summary, with awareness of the technical resolution constraint in discriminating individual bursts with a burst cluster, the data appear to support size modulation intrinsically and perfectly support frequency modulation.

## 4. Discussion

In response to signals from the cellular cascade pathways, the nuclear abundance of transcriptional activators changes, followed by changes in the transcriptional activities of related genes. Given the discovery that transcription generally occurs in discontinuous bursts, we questioned how the activators modulate bursting dynamics and how the bursting transmits information. In principle, both burst size and frequency are possible regulatory targets, and indeed, there appeared to be evidence for both scenarios [5,7,15,16,21,22,23].

Experimentally, it is hard to ascertain whether the burst size, frequency, or both are subject to modulation. This is due to technical difficulties in differentiating individual transcription initiation events. While manipulating with a time resolved fluorescence intensity curve that represents the dynamics of transcriptional bursting, it is inevitable to define artificial detection thresholds in quantifying burst size and magnitude [2,4,7,8,24,25,26,27,28,29,30]. In particular, it is even impossible to define a rational threshold for differentiating overlapping bursts. Such resolution constraints potentially lead to incorrect conclusions, rendering the debate on burst modulation an open problem.

Noting that transcriptional uncertainty is a manifestation of transcriptional dynamics, we thus collected those data that reflected intrinsic transcriptional uncertainty rather than extrinsic noise or methodology. The common characteristics of transcriptional uncertainty imply that the bursting dynamics are likely modulated by the same way. This inspired us to investigate the burst modulation with the use of two theoretical methods that independently explored such uncertainty data from the cell population measurement. Such a strategy takes advantage of bypassing the technical resolution constraints.

Both the theoretical methods are classic. One is the master equation modeling of the two-state model [36]. The two-state model is the most widely used model for describing gene expression. Although simple, it does not lose generality and is analytically tractable, while sophisticated multi-state models tend to be gene-specific and contain too many parameters. Meanwhile, the two-state model is somehow too simplistic for an in-depth investigation. We thus introduced the other method, Shannon’s information entropy investigation. The information entropy naturally characterizes the uncertainty of the signal transmission. Here, the required probability of a transcription initiation event to occur is derived based on the general architecture of the transcriptional apparatus.

Through comparing the results by theoretical investigations with experimental data, we showed that transcriptional regulation is achieved via modulating burst frequency. Investigations based on the two-state model have shown that cellular signaling benefit from low noise if transcriptional regulation is by modulating burst frequency rather than size, and that only frequency modulation presents the correct intrinsic noise of gene expression. Based on the known molecular mechanism of transcription, the information entropy of transcriptional regulation reproduces experimental data from different promoters, suggesting that burst frequency encodes regulatory information obeying a probability function. This also suggests that “frequency modulation” and “time fraction of functional enhancer occupation” are equivalent expressions of transcriptional regulation. We further demonstrate that, taking into account the existence of burst clusters that can be mistaken as individual bursts of large size, the data supporting size modulation intrinsically support frequency modulation. This work thus provides a unified scheme that reconciles the burst debate, independent of technical resolution constraint.

## 5. Conclusions

In summary, transcription signals in a “digital” way, supported by two independent theoretical investigations that bypass technical resolution constraints. A higher activator level is followed by a larger burst frequency, whereas the burst size remains unchanged. The increase in burst frequency is due to the increase in the time fraction of enhancer occupation by activators, whereas the observed burst size increase is actually a manifestation of overlapping bursts. This work thus provides a unified scheme that reconciles the debate on burst signaling.
